# Considerations for Pairing Cognitive Behavioral Therapies and Non-invasive Brain Stimulation: Ignore at Your Own Risk

**DOI:** 10.3389/fpsyt.2021.660180

**Published:** 2021-04-12

**Authors:** Christine A. Conelea, Suma Jacob, A. David Redish, Ian S. Ramsay

**Affiliations:** ^1^Department of Psychiatry and Behavioral Sciences, University of Minnesota, Minneapolis, MN, United States; ^2^Department of Neuroscience, University of Minnesota, Minneapolis, MN, United States

**Keywords:** neuromodulation, cognitive behavioral therapy, intervention, brain stimulation, neuroscience

## Abstract

Multimodal approaches combining cognitive behavioral therapies (CBT) with non-invasive brain stimulation (NIBS) hold promise for improving the treatment of neuropsychiatric disorders. As this is a relatively new approach, it is a critical time to identify guiding principles and methodological considerations to enhance research rigor. In the current paper, we argue for a principled approach to CBT and NIBS pairings based on synergistic activation of neural circuits and identify key considerations about CBT that may influence pairing with NIBS. Careful consideration of brain-state interactions and CBT-related nuances will increase the potential for these combinations to be positively synergistic.

## Introduction

A paradigm shift in research focused on neuropsychiatric applications of non-invasive brain stimulation (NIBS) is underway. The traditional focus on non-invasive brain stimulation (NIBS) monotherapy has shifted to calls for research coupling NIBS with cognitive and behavioral interventions (1, 2), reflecting findings of the past two decades demonstrating NIBS effects are “state dependent”: stimulation outcomes depend upon the state of neural activity in the targeted cortical region (3). Recognition of this interaction has sparked interest in improving NIBS efficacy via “functional targeting” that combines NIBS with cognitive tasks that modulate the same circuit being stimulated (4).

One functional targeting approach for psychiatric applications has been to combine NIBS with cognitive-behavioral therapies (CBT). “CBT” encompasses therapy procedures that target maladaptive behaviors and cognitions that underlie psychopathology. CBT is a logical choice for NIBS augmentation. Broadly speaking, CBT has both a strong evidence base and room to be enhanced in terms of efficacy, efficiency, durability, and impact on symptom improvement. CBT enables some degree of control over brain state, and research on the neural mechanisms of CBT is increasingly informing our understanding of its effects on the brain. Early research in this area suggests that combined CBT+NIBS protocols may enhance patient outcomes (5, 6).

We contend that launching this research necessitates we (1) follow principled approaches to inform decisions about *how* to combine CBT and NIBS, (2) identify key CBT considerations that may influence the rigor of future research, and (3) leverage insights about these assumptions into novel methodologies. In the current paper, we highlight several key considerations related to combining therapist-delivered CBT with NIBS techniques that can be feasibly administered simultaneously or nearly-simultaneously with CBT (i.e., transcranial magnetic stimulation, TMS; transcranial electrical stimulation, tES).

## Successful CBT+NIBS Interventions Depend Upon Neural Circuit Matchmaking

Functional targeting requires that NIBS and the behavior elicited by a CBT procedure synergistically engage neurocircuitry. It is well-established that different, clinically relevant behaviors targeted in CBT arise from information processing within different neural circuits (7–9). For example, fear conditioning accesses amygdala circuits (10), and override processes that allow behaviors to proceed in spite of fear access ventromedial prefrontal regions [IL in rats (11, 12) and subgenual ACC in humans (13)]. Planning processes depend on prefrontal-hippocampal circuits (14), overtrained habit processes depend on circuits between motor cortical regions and dorsolateral striatum (15), and motivation and reward processes depend on orbitofrontal, ventral tegmental, and nucleus accumbens circuits (16). Current theories suggest that psychiatric conditions can arise from multiple dysfunctions within these neural circuits, and that treatment will need to be focused on repairing damaged circuits or enhancing compensating circuits (17).

CBT+NIBS interventions should activate common or complementary circuitry (2), or otherwise engage compensatory circuits to enhance CBT outcomes. Eliciting specific thoughts, memories, and action-selection processes subserved by the aforementioned circuits through behavioral techniques (such as CBT) makes them labile and manipulable (18–20). This privileges those thoughts and actions to modification, suggesting that the sensitivity of neural circuits will depend on their activation. This suggests a way forward whereby specific CBT-elicited behavioral interactions activate certain circuits, making them amenable to targeted manipulation by neuromodulation techniques. This also implies that NIBS protocols should be designed to bias a circuit engaged by a CBT-evoked behavior toward the desired outcome (e.g., by increasing or decreasing circuit activity). Empirical testing is needed to clarify optimal CBT+NIBS pairings—the key is to begin testing pairings based on hypothesized synergistic co-activation of neural circuits.

## Considerations for Future Research

### Consideration 1: CBT Is a Collection of Heterogeneous, Dynamic Interventions, and Does Not Uniformly Engage Single Neural Circuits

CBT interventions have shared characteristics (21) but are organized into specific protocols that target particular diagnoses or transdiagnostic processes, developmental stages, patient groups, and/or practice settings. CBT protocols are, by design, multi-component interventions. Components include procedures that target specific symptomatology and those that enhance therapy uptake or durability. Content is intentionally dynamic to support learning and often individualized to address idiographic symptom presentation. Multiple components are also typically delivered within a single therapy session. Notably, there is ongoing debate about which components are necessary and sufficient within particular CBT protocols (22, 23), and the precise learning processes and neural circuits that individual CBT elements impact are not entirely known (24).

Because CBT is a heterogeneous, dynamic intervention, it does not uniformly engage single neural circuits. Optimal CBT+NIBS pairing depends on understanding “*which circuits are engaged when*.” Future research must develop dynamic functional targeting approaches that enable optimal NIBS delivery and timing depending on the specific CBT elements engaged per session. We must identify the neural circuitry driving a behavioral output before NIBS can be used to modulate the circuitry supporting the targeted behavior. Methodological details that enhance our fine-grained understanding of CBT+NIBS pairings and enhance replicability should be included in published protocols. Though common practice, identifying an intervention only as “CBT” is like calling a specific pharmaceutical a “medication.” CBT+NIBS trials should specify the exact protocol used and detail timing and duration of procedural elements within CBT sessions and in relation to stimulation.

### Consideration 2: CBT+NIBS Synergy May Not Necessarily Result From Stimulating a Circuit Shown to Change Pre-post CBT

Due to CBT's dynamic nature, the ways that specific CBT+NIBS procedures interact may be inconsistent over time. For example, circuits are not necessarily engaged consistently within and across CBT sessions and can differ depending on learning stage (25). There may also be individual differences in the circuits patients engage to arrive at the same clinical outcomes, as well as a combination of restorative and compensatory mechanisms associated with treatment response. Animal models may provide insights into how circuit engagement is influenced by biological therapeutics (e.g., stimulation, medication), behavioral training, and potential moderators (e.g., genetics, learning history, development, sex/hormonal status), as well as highlight individual differences to leverage and personalize CBT+NIBS. We should also consider strategies to time-lock circuit-based measurement with methods that quantify human behavior or targeted neural activation during CBT procedures in an effort to inform closed-loop neuromodulation (26). One emerging technique that may be useful in this regard is brain oscillation-synchronized TMS, which uses real-time electroencephalography (EEG) to trigger TMS pulses depending on the oscillatory phase of the EEG signal (27).

### Consideration 3: Delivering a Procedural Element of CBT Is Not Equivalent to Delivering a Full CBT Protocol

Some approaches to combining CBT and NIBS have delivered a procedure from within a CBT protocol alongside stimulation, such as presenting anxiety cues as a proxy for exposure therapy (28, 29). This approach may be useful, in that it may more selectively engage a behavior/circuit. However, this approach becomes problematic when critical elements of the procedure are discarded. For example, in trials presenting anxiety cues for OCD (28) and PTSD (29), elements necessary for corrective learning from exposure were not included [e.g., activation of anxiety, restriction of avoidance/escape behaviors (30, 31)]. This example highlights the problem of plucking a procedure out of CBT without attending to specific procedural details that render it therapeutic. A CBT procedure labeled as “therapy” should contain all procedural elements known to be critical for therapeutic change. Methodological decisions about which CBT components to keep or discard alongside NIBS must consider the broader theory and evidence base underpinning the CBT intervention. If a NIBS study uses a CBT component outside of a full CBT package, the element selected should be described precisely (e.g., “anxiogenic stimulus presentation” instead of “exposure therapy”), and a rationale for this choice and implementation should be provided. If participants are given choices about how to engage in the procedure, engagement should be explored as a moderator of outcomes.

### Consideration 4: CBT Efficacy Varies Across Individuals and Practitioners

Though CBT is a class of effective interventions with solid empirical support, effects are generally in the “medium” range and vary by disorder (32, 33). Individual differences in personality, motivation, psychosocial environment, cognitive ability, genetics, and neural processes influence CBT gains (34–37). Specific CBT interventions can have unique mediators and moderators of response that may limit or enhance efficacy. Therapist factors can also impact outcomes, such as clinician competence, training, theoretical orientation, protocol adherence, and personal characteristics. Failure to impact clinical outcomes in CBT+NIBS trials may not be a shortcoming of NIBS, but instead reflect CBT's variable efficacy. Efficacy of the standalone CBT protocol should be demonstrated prior to NIBS augmentation. CBT+NIBS trials should also incorporate established treatment fidelity methods to ensure that the CBT is delivered, received, and enacted as intended (38, 39). Quantification of process elements [e.g., patient/therapist behaviors (40)] and measurement of relevant moderators and mediators should also be considered, as these methods may reveal causes of variable outcomes, information that can in turn be used to further refine, personalize, or optimize the intervention.

### Consideration 5: The Change Agent of CBT Often Occurs Outside the CBT Session

A core feature of CBT protocols is the completion of “homework” outside of the formal therapy session. Homework typically entails skills practice for learning and generalization, and it engages therapeutic mechanisms necessary for clinical change (41). While some in-session CBT components, such as *in vivo* exposure, do activate therapeutic mechanisms, homework to repeatedly engage these mechanisms is seen as crucial for solidifying learning and ensuring that gains are not specific to the clinic context (42). Furthermore, in some CBT protocols, the majority or entire therapeutic change process is presumed to occur outside of session, such that the session itself is used to plan and prepare for homework (43). Homework completion is an important predictor of treatment response (44, 45), underscoring that some essential aspects of CBT occur outside of the clinic. This poses challenges for CBT+NIBS, such that neuromodulation may not be delivered in conjunction with mechanisms driving therapeutic change.

To impact homework (or the mechanisms engaged by homework), NIBS likely needs to be deployed in close temporal proximity to skills practice or delivered in naturalistic circumstances. One approach could be to adapt CBT sessions to emphasize active skills implementation or rehearsal concurrently or sequentially to stimulation. Optimal timing of specific CBT and NIBS procedures should be tested; although concurrent administration seems preferable for tES (2), timing is more of an open question for TMS and may differ depending on the outcome being targeted. Another approach could be to test use of NIBS to target circuitry that underlies skill acquisition (learning) during a CBT session. Finally, making NIBS more accessible in a patient's natural environment could enable pairing of homework with stimulation, as well as potentially offer the added benefit of enhancing skills generalization across contexts. Home-based tES delivery holds promise in this regard. Existing research demonstrates home-based tES is acceptable and safe, and guidelines for facilitating compliance and safety monitoring have been established (46, 47).

### Additional NIBS Considerations

It is beyond the scope of this paper to review the progress and challenges of NIBS to date, but here we highlight a few NIBS-specific considerations relevant to pairing it with CBT. First, individual patients can respond differently to the same NIBS procedures, with variable response attributable to many factors, including brain anatomy and physiology, medications, and hormonal status. Efforts to control, measure, and ultimately tailor NIBS protocols [e.g., to subgroups or biotypes (48)] should also be considered in CBT augmentation research.

Second, NIBS outcomes reflect an interaction between brain state and the modulated circuit. Cognitive processing, concurrent behavior, emotional state, priming, and wakefulness can moderate NIBS effects (49). Unfortunately, research often overlooks the importance of brain state in favor of focusing on technical aspects of NIBS delivery (e.g., biomechanics of device). Protocols often specify only stimulation parameters and the cortical region being targeted. While these factors are critical to study, doing so without considering brain state attends to only “half of the equation.” Our understanding of CBT+NIBS would be greatly improved by research that systematically measures and manipulates brain states alongside circuit modulation.

Third, more research is needed to determine how different NIBS methods impact specific circuits acutely and longitudinally. Pre-clinical and translational research that systematically and parametrically tests how particular NIBS methods impact disease- or CBT-relevant circuits is a useful prerequisite to informing CBT+NIBS pairings. For example, systematic translational studies can be used to optimize stimulation parameters prior to deploying the NIBS as a treatment [e.g., see (50) for an example in TMS for cocaine use disorder]. Research focused on testing how to best time NIBS delivery in relation to CBT protocols is also critically needed. Timing parameters that could be explored include simultaneous delivery, near-simultaneous delivery (e.g., one immediately following the other, where the first primes the targeted circuit), or sequential delivery (e.g., fully completing one intervention before the other).

Fourth, we should not assume that the circuit that is dysfunctional in a given disorder is the right one to stimulate alongside CBT. As noted above, optimal pairing likely depends on stimulating circuits that promote CBT-evoked behaviors. It is reasonable to suspect that these may in some situations be different circuits than those driving pathology. Researchers should also consider targeting circuits that engage compensatory processes or enhance cognitive strengths. If we over-focus on targeting deficits in an attempt to “normalize” functioning, we will miss opportunities to leverage patient strengths that can improve clinical functioning.

## Discussion

The convergence of CBT and NIBS research presents promising opportunities to improve the well-being of those living with psychiatric illness, though we must proceed thoughtfully. If the goal of combining NIBS and CBT is to improve patient outcomes, we have to carefully consider brain state-circuit interactions, circuit activity during specific CBT components, timing of stimulation, and the influences of individual differences, providers, and delivery format. Quantifying what actually happens in and out of CBT sessions will help identify optimal ways to arrange positive synergy between both modalities. We can use NIBS to either target the neural processes that benefit from that CBT component *or* boost compensatory processes to enhance benefit from that component.

We should also consider innovative ways to modify CBT to better work alongside NIBS. CBT could be modified to more precisely and effectively target neurocognitive processes that are most likely to drive clinical change, for example by dropping unnecessary CBT components or changing the process of how the CBT component is delivered. CBT approaches that target specific symptomatology or cognitive endophenotypes with known underlying circuitry may also be better candidates for NIBS augmentation than CBT approaches that target DSM diagnoses or general cognitive or emotional processes.

These considerations generalize to other multimodal intervention approaches. New technologies that directly manipulate neural circuits are continually emerging. Combining new technologies with CBT will require cognizance of which neural circuits are impacted by CBT so that these two paradigms will be synergistic rather than passing by each other, or worse, interfering with each other.

## Data Availability Statement

The original contributions presented in the study are included in the article/supplementary material, further inquiries can be directed to the corresponding author/s.

## Author Contributions

CC developed the paper concept and contributed to writing and editing. SJ, AR, and IR contributed to refining the paper concept and contributed to writing and editing. All authors contributed to the article and approved the submitted version.

## Conflict of Interest

CC and SJ are investigators on National Institute of Mental Health-funded SBIR grants awarded to Posit Science [R43MH121209 (CC), R43MH124542 (CC and SJ)]. SJ has been an investigator in multisite autism treatment trials by Roche and on an autism advisory board for Roche. The remaining authors declare that the research was conducted in the absence of any commercial or financial relationships that could be construed as a potential conflict of interest.
